# An Observational Study of Trifluridine/Tipiracil-Containing Regimen Versus Regorafenib-Containing Regimen in Patients With Metastatic Colorectal Cancer

**DOI:** 10.3389/fonc.2022.867546

**Published:** 2022-05-19

**Authors:** Meng-Che Hsieh, Kun-Ming Rau, Shung-Eing Lin, Kuang-Wen Liu, Chong-Chi Chiu, Chih-I Chen, Ling-Chiao Song, Hsin-Pao Chen

**Affiliations:** ^1^ Department of Hematology-Oncology, E-Da Cancer Hospital, Kaohsiung, Taiwan; ^2^ College of Medicine, I-Shou University, Kaohsiung, Taiwan; ^3^ Division of Colon and Rectum Surgery, Department of Surgery, E-Da Cancer Hospital, Kaohsiung, Taiwan; ^4^ Division of Colon and Rectum Surgery, Department of Surgery, E-Da Hospital, Kaohsiung, Taiwan; ^5^ Division of General Surgery, Department of Surgery, E-Da Cancer Hospital, Kaohsiung, Taiwan

**Keywords:** metastatic colorectal carcinoma, trifluridine/tipiracil, regorafenib, oncologic outcome, prognosis

## Abstract

**Background:**

There are no randomized control trials comparing the efficacy of trifluridine/tipiracil and regorafenib in patients with metastatic colorectal cancer (mCRC). Herein, we conducted an observational study to compare the oncologic outcomes of trifluridine/tipiracil-containing regimen (TAS-102) and regorafenib-containing regimen (REG) in patients with mCRC.

**Material and method:**

Patients who were diagnosed to have mCRC in 2015 to 2021 and treated with TAS-102-containing regimen or REG-containing regimen were recruited. Monotherapy or combination therapy were all allowed in this study. Oncologic outcomes were presented with progression-free survival (PFS), overall survival (OS), overall response rate (ORR) and disease control rate (DCR).

**Results:**

A total of 125 patients were enrolled into our study, accounting for 50 patients with TAS-102 and 75 patients with REG. Of these patients, 64% were treated with TAS-102 or REG monotherapy, while the remaining were treated with TAS-102 combination or REG combination. In general, the median PFS and OS were 3.7 versus 2.0 months (*P* = 0.006) and 9.2 versus 6.8 months (*P* = 0.048) in TAS-102 and REG, respectively. The ORR and DCR were 44% versus 20% (*P* < 0.001) and 72% versus 43% (*P* < 0.001) in TAS-102 and REG, respectively. As for treatment strategies, the survival were significantly longer in combination than in monotherapy, no matter in TAS-102 or REG group. Multivariate analysis showed TAS-102 and combination therapy were independent predictor associated with better survival.

**Conclusions:**

Our results suggested that TAS-102 had better oncologic outcomes than REG in patients with mCRC, especially in combination. Further prospective trials are warranted to confirm our results.

## Introduction

Colorectal cancer (CRC) is one of the most common gastrointestinal tract cancers in nowadays. It is the third prevalent malignancy and the second leading cause of cancer-related death worldwide. There are more than 1.9 million new patients diagnosed to have CRC and 935,000 deaths attributed to CRC in 2020 (1). Among these patients, more than half developed metastatic colorectal cancer (mCRC) eventually. The standard therapies for patients with mCRC includes chemotherapy regimens containing irinotecan, oxaliplatin, and fluoropyrimidines in combination with anti-vascular endothelial growth factor (VEGF) or anti-epidermal growth factor receptor (EGFR) antibodies (2). With these treatments, the overall survival of mCRC has improved gradually with an estimated median survival about 30 months and 5-year survival rate about 14% (3). However, the prognosis of mCRC drops sharply when in chemorefractory status. The median survival after chemorefractory is approximately 6 months. Hence, there is an urgent need to improve outcomes in patients with chemorefractory mCRC.

For chemorefractory mCRC, two oral agents, trifluridine/tipiracil (Lonsurf; TAS-102; Taiho Pharmaceutical, TTY Biopharm., Taiwan) and regorafenib (REG; Bayer AG, Berlin, Germany), have been proved as third to fourth-line treatment to prolong survival. TAS-102 is composed of an antineoplastic thymidine-based nucleoside analog (trifluridine) and an inhibitor of thymidine phosphorylase that degrades trifluridine (tipiracil) (4). The pivotal phase 3 RECOURSE trial compared TAS-102 with placebo in patients with refractory mCRC and demonstrated that TAS-102 significantly prolonged overall survival (OS) (7.1 months vs. 5.3 months, p < 0.001) and progression-free survival (PFS) (2.0 months vs. 1.7 months, p < 0.001) as compared with placebo (5). REG is an oral multi-kinase agent that inhibits activity of several stromal receptor tyrosine kinases associated with angiogenesis, oncogenesis, and the tumor microenvironment (6). The pivotal phase 3 CORRECT trial compared REG and placebo in patients with refractory mCRC and demonstrated that REG resulted in significantly longer OS (6.4 months vs. 5.0 months, p: 0.0052) and PFS (1.9 months vs. 1.7 months, p< 0.0001) as compared with placebo (7). Based on these results, both TAS-102 and REG gain the indication of refractory mCRC. Current guidelines also indicate that TAS-102 and REG are both effective regimens in later-line treatment of mCRC (2).

Nonetheless, there are no randomized control trials directly comparing the efficacy of TAS-102 and REG. Previous retrospective studies had published their prognosis of TAS-102 monotherapy and REG monotherapy. Kawakami et al. analyzed a nationwide database in which TAS-102 demonstrated significantly longer survival than REG (8), while other publications showed insignificant survival between TAS-102 and REG (9, 10). Moreover, recent evidences exhibited combination strategy of TAS-102 and REG with longer survival benefits numerically (11, 12). However, no comprehensive studies focused on the comparison between TAS-102 combination and REG combination. Given the inconclusive results, we conducted an observational study to compare the oncologic outcomes of TAS-102-containing regimen and REG-containing regimen in patients with mCRC.

## Materials and Methods

### Patients

Patients who were at the age older than 18 years and diagnosed with pathologically proved mCRC from 2015 to 2021 at E-Da Hospital and E-Da Cancer Hospital were retrospectively reviewed. Patients who failed at least 2 line of standard chemotherapy and treated with TAS-102 or REG as later line treatment were enrolled into our study. Standard chemotherapy includes oxaliplatin, irinotecan, 5-fluorouracil, anti-VEGF antibody and anti-EGFR antibody (if RAS wild type). All the patients’ basic characteristics were retrieved from medical records. Exclusion criteria were previous history of other cancer, irregular evaluation intervals and lost follow-up. This was a retrospective observational study, which was exempt from requiring consent. This study was approved by the E-Da Hospital Institutional Review Board (EMPR-109-012), and was conducted in accordance with the Declaration of Helsinki.

### Treatments

REG was administered orally with an initial dose of 160mg daily on days 1–21 with 7 days of rest. TAS-102 was administered orally with an initial dose of 35 mg/m2 twice daily for 5 days a week with 2 days of rest for 2 consecutive weeks, followed by 14 days of rest. Both drugs were repeated every 4 weeks. Combination treatment includes anti-VEGF targeted therapy, anti-EGFR targeted therapy, oxaliplatin or irinotecan. Dose modification could be adjusted at physician’s discrete based on patients’ comorbidities and treatment adverse effects. Computed tomography was evaluated for the treatment response every 2-3 months. The treatments were continued in responding or stable patients until tumor progression, death or intolerable toxicities.

### Statistical Analysis

All the basic characteristic were retrospectively retrieved from a medical chart review and presented with frequencies. Chi-square tests were calculated to analyze the differences between TAS-102 and REG. Statistical analyses were performed using SPSS. Oncologic outcomes were presented with progression-free survival (PFS), overall survival (OS), overall response rate (ORR) and disease control rate (DCR). Progression-free survival (PFS) was measured from the first day of chemotherapy administration until the date of tumor progression or final follow-up, while overall survival (OS) was calculated as the time from the first day of chemotherapy administration until the date of death from any cause or final follow-up. Objective response criteria in the tumors, including complete response (CR), partial response (PR), stable disease (SD), and progressive disease (PD), were evaluated according to the RECIST 1.1 guidelines. ORR was defined as CR plus PR, and DCR was defined by CR, PR, plus SD. Kaplan–Meier curves were depicted for survival. We also conducted Cox regression analysis using “enter” selection to adjust for the effects of potential confounders. All P values were two sided and considered to have significance if *P* values < 0.05.

## Results

### Patients Characteristics

A total of 125 patients were enrolled into our study for oncologic outcomes evaluation with a median follow-up period 20 months. The median age of our patients is 64 years. Baseline characteristics were presented in **Table 1**. In general, most patients were male in gender (56%) and older than 60 years (70%). The majority of primary tumor location was left side colon (78%). Nearly 90% of our patients had initial stage 3-4 disease, 78% received radical surgery and 62% underwent adjuvant chemotherapy. As for genetic profiles, 62% of patient had all RAS mutant, 98% were B-raf wild type and 99% were MMR proficient. The median time from diagnosis of metastases to enroll into our study was 18.5 months. Most patients received TAS-102 or REG as fourth-line treatment. As for treatment strategies, 64% patients were treated with TAS-102 or REG monotherapy, while the remaining were treated in combination with other agents, including targeted therapy or chemotherapy. After stratified by chemotherapy, 50 patients received TAS-102 and 75 patients received REG for their chemorefractory mCRC. In TAS-102 group, 52% patients received TAS-102 monotherapy and 48% received TAS-102 combination therapy. In TAS-102 combination group, 60% patients was treated in combination with anti-VEGF agents rechallenge and 40% in combination with anti-EGFR agents rechallenge. In REG group, 72% patients received REG monotherapy and 28% received REG combination therapy. In REG combination group, 60% patients were treated in combination with irinotecan rechallenge and 40% were treated in combination with oxaliplatin rechallenge. All basic characteristics including gender, age, primary tumor location, initial stage, previous history, genetic status, time from diagnosis of metastases and number of prior regimens were well balanced between the two treatment arms.

**Table 1:** Baseline characteristics of patients with mCRC treated with TAS-102 or REG.

	TAS-102	REG	p
	N =50	N = 75	
Gender			0.585
Male	26 (52%)	44 (59%)	
Female	24 (48%)	31 (41%)	
Age			0.602
≦ 60	16 (32%)	21 (28%)	
> 60	34 (68%)	54 (72%)	
Primary tumor location			0.131
Right side colon	14 (28%)	14 (19%)	
Left side colon	36 (72%)	61 (81%)	
Initial stage			0.967
Stage 1-2	6 (12%)	9 (12%)	
Stage 3-4	44 (88%)	66 (88%)	
Previous surgery			0.253
Yes	42 (84%)	56 (75%)	
No	8 (16%)	19 (25%)	
Previous adjuvant chemotherapy			0.683
Yes	32 (64%)	46 (61%)	
No	18 (35%)	29 (39%)	
All ras status			0.582
Mutant type	32 (64%)	45 (60%)	
Wild type	18 (36%)	30 (40%)	
B-raf status			0.901
Mutant type	1 (2%)	2 (3%)	
Wild type	49 (98%)	73 (97%)	
MMR status			0.185
Proficiency	49 (98%)	75 (100%)	
Deficiency	1 (2%)	0 (0%)	
Time from diagnosis of metastases			0.795
≦ 18 weeks	22 (44%)	35 (47%)	
> 18 weeks	28 (56%)	40 (53%)	
Number of prior regimens			0.332
2	20 (40%)	25 (33%)	
3	22 (44%)	40 (53%)	
4	8 (16%)	10 (14%)	
Treatment strategy			0.042
Monotherapy	26 (52%)	54 (72%)	
Combination	24 (48%)	21 (28%)	

mCRC, metastatic colorectal cancer; TAS-102, trifluridine/tipiracil; REG, regorafenib; MMR, mismatch repair.

### Survival Outcomes

The oncologic outcomes between TAS-102 and REG were summarized in **Table 2**.

**Table 2:** Oncologic outcomes of 125 patients with mCRC treated with TAS-102 and REG.

	TAS-102				REG			
	Total	Combo	Mono	*P*	Total	Combo	Mono	*P*
N	50	24	26		75	21	54	
mPFS (m)	3.7	6.6	2.0	< 0.001	2.0	4.8	1.8	< 0.001
mOS (m)	9.2	16.7	6.5	0.008	6.8	14.5	4.9	< 0.001
CR (%)	0%	0%	0%		0%	0%	0%	
PR (%)	22 (44%)	17 (71%)	4 (15%)		15 (20%)	10 (48%)	5 (9%)	
SD (%)	14 (28%)	5 (21%)	10 (39%)		17 (23%)	7 (33%)	10 (19%)	
PD (%)	14 (28%)	2 (8%)	12 (46%)		43 (57%)	4 (19%)	39 (72%)	
ORR (%)	44%	71%	15%	< 0.001	20%	48%	9%	< 0.001
DCR (%)	72%	92%	54%	0.007	43%	81%	28%	< 0.001

mCRC, metastatic colorectal cancer; TAS-102, trifluridine/tipiracil; REG, regorafenib; mPFS, median progression-free survival; mOS, median overall survival; CR, complete response; PR, partial response; SD, stable disease; PD, progressive disease; ORR, objective response rate; DCR, disease control rate.

**Figure 1:**
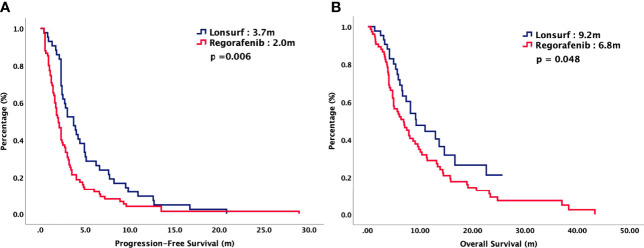
**(A)** Progression-free survival and **(B)** overall survival of 125 patients with chemorefractory mCRC, stratified by chemotherapy regimen.

**Figure 2:**
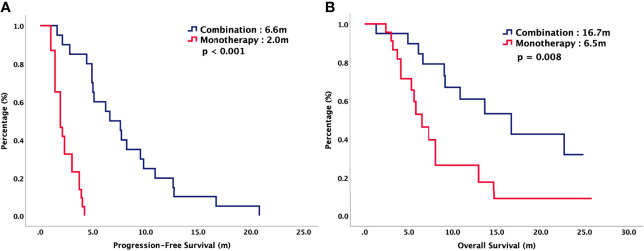
**(A)** Progression-free survival and **(B)** overall survival of patients with chemorefractory mCRC treated with TAS-102, stratified by combination or monotherapy.

**Figure 3:**
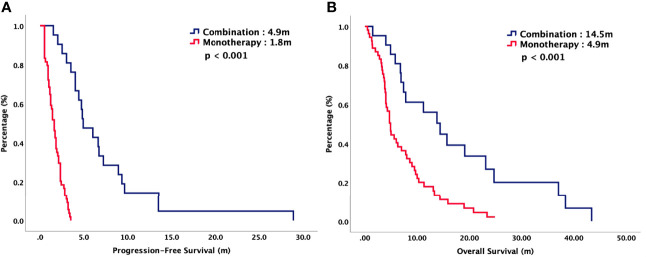
**(A)** Progression-free survival and **(B)** overall survival of patients with chemorefractory mCRC treated with REG, stratified by combination or monotherapy.

For total population, the median PFS were 3.7 months in TAS-102 and 2.0 months in REG (*P* = 0.006). The median OS were 9.2 months in TAS-102 and 6.8 months in REG (*P* = 0.048). The ORR and DCR were 44% versus 20% (*P* < 0.001) and 72% versus 43% (*P* < 0.001) in TAS-102 and REG, respectively. The survival curves of PFS and OS are plotted in **Figure 1**. Moreover, all patients were divided according to combination or monotherapy. As for treatment strategy, the survival is significantly longer in combination than those in monotherapy, no matter in TAS-102 or REG group. The survival curves of PFS and OS of TAS-102 stratified by treatment strategy were plotted in **Figure 2**. For patients treated with TAS-102, the median PFS and OS were 6.6 months versus 2.0 months (*P* < 0.001) and 16.7 months versus 6.5 months (*P* < 0.001) in combination and monotherapy groups, respectively. For patients treated with REG, the median PFS and OS were 4.8 months versus 1.8 months (*P* < 0.001) and 14.5 months versus 4.9 months (*P* < 0.001) in combination and monotherapy groups, respectively. The survival curves of PFS and OS of REG stratified by treatment strategy were plotted in **Figure 3**.

### Multivariate Regression Analysis

Cox regression analyses with survival for potential prognostic factors were performed. Hazzard ration (HR) with 95% CIs were depicted in **Table 3**. Univariate analysis showed initial stage (HR: 0.56, 95% CI: 0.36-0.88, P = 0.011 for PFS, HR: 0.50, 95% CI: 0.31-0.81, P = 0.005 for OS), chemotherapy regimen (HR: 0.62, 95% CI: 0.42-0.91, P = 0.014 for PFS, HR: 0.60, 95% CI: 0.40-0.98, P = 0.040 for OS) and treatment strategy (HR: 0.07, 95% CI: 0.03-0.14, *P* = <0.001 for PFS, HR: 0.35, 95% CI: 0.22-0.56, *P* = <0.001 for OS) were strongly correlated with PFS and OS. Multivariate analysis demonstrated that TAS-102 (HR: 0.63, 95% CI: 0.42-0.93, P = 0.020 for PFS, HR: 0.60, 95% CI: 0.37-1.00, P = 0.050 for OS) and combination (HR: 0.07, 95% CI: 0.03-0.15, P = <0.001 for RFS, HR: 0.38, 95% CI: 0.24-0.62, P = <0.001 for OS) were independent predictor associated with better survival.

**Table 3:** Cox regression analysis of parameters associated with survival in mCRC patients treated with TAS-102 or REG.

	PFS				OS				
	Univariate		Multivariate		Univariate		Multivariate		
Variables	HR (95% CI)	*P* value	HR (95% CI)	*P* value	HR (95% CI)	*P* value	HR (95% CI)	*P* value
Gender, female vs. male	0.83 (0.57-1.20)	0.321			0.90 (0.59-1.36)	0.609		
Age, ≦ 60 vs. > 60	0.96 (0.64-1.43)	0.824			0.79 (0.48-1.28)	0.337		
Primary tumor location, right side vs left side	0.97 (0.60-1.56)	0.901			0.75 (0.43-1.33)	0.330		
Initial stage, 1-2 vs. 3-4	0.56 (0.36-0.88)	0.011	0.78 (0.50-1.22)	0.275	0.50 (0.31-0.81)	0.005	0.77 (0.48-1.23)	0.277
Previous surgery, yes vs. no	0.86 (0.49-1.51)	0.594			0.53 (0.26-1.26)	0.170		
Previous adjuvant chemotherapy, yes vs. no	0.73 (0.49-1.06)	0.100			0.79 (0.51-1.21)	0.279		
All ras status, wild type vs. mutant type	0.89 (0.61-1.30)	0.556			0.95 (0.61-1.45)	0.797		
B-raf status, wild type vs. mutant type	0.58 (0.18-1.85)	0.369			0.56 (0.11-1.35)	0.184		
MMR status, deficiency vs. proficiency	0.34 (0.05-2.45)	0.283			0.15 (0.01-3.51)	0.461		
Time from diagnosis of metastases. >18 vs. ≦ 18 weeks	0.74 (0.51-1.07)	0.113			0.80 (0.52-1.22)	0.294		
Number of prior regimens, 2 vs. 3-4	0.66 (0.31-1.22)	0.759			0.73 (0.28-1.31)	0.882		
Chemotherapy regimen, TAS-102 vs. REG	0.62 (0.42-0.91)	0.014	0.63 (0.42-0.93)	0.020	0.63 (0.40-0.98)	0.040	0.60 (0.37-1.00)	0.050
Treatment strategy, combo vs. monotherapy	0.07 (0.03-0.14)	<0.001	0.07 (0.03-0.15)	<0.001	0.35 (0.22-0.56)	<0.001	0.38 (0.24-0.62)	<0.001

mCRC, metastatic colorectal cancer; TAS-102, trifluridine/tipiracil; REG, regorafenib; PFS, progression-free survival; OS, overall survival; HR, hazard ratio; CI, confidence interval; MMR, mismatch repair gene.

## Discussion

To our best knowledge, this is the first study to demonstrate that combination is much better than monotherapy in mCRC patients treated with TAS-102 or REG. Previous literature all focused on the comparison between TAS-102 monotherapy and REG monotherapy. The phase 3 RECOURSE trial demonstrated that TAS-102 significantly prolonged survival (5) and the phase 3 CORRECT trial also demonstrated that REG resulted in significantly longer survival (7). The PFS was increased 0.3 months and the OS was increased 1.4 - 1.8 months. Although these two studies are significant, the survival differences were modest numerically. Moreover, Kuboki et al. conducted a phase 1/2 C-TASK FORCE trial to analyze the efficacy of TAS-102 in combination with bevacizumab (11). This study suggested that TAS-102 plus bevacizumab combination might become a potential treatment option in chemorefractory mCRC patients, with median PFS 5.6 months and median OS 11.4 months. Another phase Ib NIVOREGO trial also showed the combination of regorafenib plus nivolumab had a manageable safety profile and encouraging antitumor activity in patients with mCRC, with ORR 36% and median PFS 7.9 m (12). Our study was consistent with these conclusions. For total population, the median PFS were 3.7 months in TAS-102 and 2.0 months in REG (*P* = 0.006). The median OS were 9.2 months in TAS-102 and 6.8 months in REG (*P* = 0.048). After stratification, the survival is significantly longer in combination than monotherapy, no matter in TAS-102 or REG group. For patients treated with TAS-102, the median PFS and OS were 6.6 months versus 2.0 months (*P* < 0.001) and 16.7 months versus 6.5 months (*P* < 0.001) in combination and monotherapy groups, respectively. For patients treated with REG, the median PFS and OS were 4.8 months versus 1.8 months (*P* < 0.001) and 14.5 months versus 4.9 months (*P* < 0.001) in combination and monotherapy groups, respectively. Multivariate analysis demonstrated that combination therapy were independent predictor associated with better survival, no matter in TAS-102 or REG. Our study demonstrated that combination therapy can achieve the best prognosis, rather than monotherapy, in chemorefractory mCRC patients treated with TAS-102 or REG. Further prospective trials are warranted to confirm with our conclusions.

Targeted therapy-chemotherapy combinations have been recognized as the optimal regimens in patients with mCRC. You et al. conducted a comprehensive meta-analysis enrolling 16 first-line clinical trials to evaluate the efficacy between chemotherapy plus targeted therapies and chemotherapy alone in mCRC patients (13). The meta-analysis suggested that the right-sided mCRC patients benefited more from chemotherapy plus bevacizumab comparing with chemotherapy alone. Arnold et al. also performed an retrospective study to compare chemotherapy plus EGFR antibody therapy with chemotherapy alone in patients with left-side mCRC (14). This study demonstrated that a greater effect of chemotherapy plus EGFR antibody therapy was observed in comparison with chemotherapy alone for patients with left-side mCRC. Our results were consistent with these conclusions that the survival is significantly longer in combination than those in monotherapy, no matter in TAS-102 or REG group. TAS-102 plus targeted therapy has a greater clinical benefit than TAS-102 alone, and REG plus chemotherapy also has a longer survival than REG alone. Immunotherapy is emerging treatment in nowadays. The indication of immunotherapy in mCRC was mainly in microsatellite instability patients. Previous studies also tested immunotherapy combination therapy in mCRC. Patel et al. conducted a phase 2 trial adding nivolumab to TAS-102 in patients with heavily pretreated microsatellite-stable (MSS) mCRC patients (15). The results showed nivolumab plus TAS-102 failed to extend clinical benefits in patients with refractory MSS mCRC. Median PFS was only 2.2 months. Another immunotherapy combination is nivolumab plus REG. Fukuoka et al. conducted a phase Ib trial of regorafenib plus nivolumab for patients with mCRC. The efficacy is promising with ORR 36% and median PFS 7.9 months. However, further prospective trials are warranted. Taking together, our study suggests that the optimal treatment strategy for mCRC patients is combination therapy like targeted therapy plus chemotherapy, rather than targeted therapy or chemotherapy alone.

Current guidelines all suggested that TAS-102 and REG are standard treatment in patients with chemorefractory mCRC (2). However, little is known about the priority and treatment sequences. Several literatures had compared the oncologic outcomes between TAS-102 monotherapy and REG monotherapy retrospectively. Chida et al. recruited 550 mCRC patients and revealed prolonged survival in patients treated with both REG and TAS-102, as compared with either REG or TAS-102 alone (16). Unseld et al. investigated the optimal treatment sequence for late-line mCRC and showed a tendency for longer PFS and OS for TAS-102 treated patients. The PFS and OS for patients treated with TAS-102 was 2.8 months and 15.9 months, respectively (17). Nakashima et al. identified 7279 patients (REG: 1501, regorafenib/TAS-102: 973, TAS-102: 3777, and TAS-102/REG: 1028) with corresponding median OS was 6.4, 16.4, 10.2, and 16.9 months, respectively. They concluded that it might be better to prescribe TAS-102 first and sequential administration of TAS-102 and REG ought to be considered because of longer OS (8). Moriwaki et al. conducted a propensity score analysis and demonstrated that no significant difference in OS between TAS-102 and REG was observed in patients with mCRC. The median OS was 7.9 months in the REG and 7.4 months in the TAS-102 (18). Ogata et al. reported a multi-institute retrospective study and found TAS-102 and REG had similar efficacy. The median OS was 9.9 and 11.4 months in the REG and TAS-102, respectively and the median PFS was 2.0 and 3.3 months in the REG and TAS-102, respectively (9). Huemer et al. performed an effectiveness and safety analysis and indicated that REG was associated with an increased hospitalization probability during palliative therapy in chemorefractory mCRC. The corresponding hospitalization probability was 30% with regorafenib versus 18% with TAS-102 at five weeks and 41% versus 28% at ten weeks, respectively (19). Patel et al. showed that mCRC patients taking TAS-102 were significantly more likely to adhere to and comply with therapy compared with those taking REG. The TAS-102 patients were 37% less likely to discontinue their treatment compared with the REG patients (20, 21). Our study was consistent with these publications that oncologic outcomes were insignificant between TAS-102 monotherapy and REG monotherapy, accounting for 2.0 vs 1.8 months, respectively. Further prospective trials with head to head comparison are ongoing.

There are several potential limitations in our work, which are inherent to any retrospective studies. Chemotherapy regimen, combination or monotherapy were decided at the discretion of physicians and patients. These might be major biases in this study. Meanwhile, a single institutional experience, a small sample size, heterogeneity of our patients and inconsistent follow-up interval may also limit the power of our study. Given that, our study first identified that combination therapy is much better than monotherapy in chemorefractory mCRC patients treated with TAS-102 or REG. Moreover, our study also confirmed that the oncologic outcomes of TAS-102 monotherapy and REG monotherapy were consistently insignificant. To date, there are no prospective randomized controlled trials with larger cohorts focusing on the comparison between TAS-102 and REG. Thus, in spite of a retrospective study with inevitable selection bias, our study remains clinically valuable.

## Conclusions

Our study investigated the oncologic outcomes of TAS-102 and REG in patients with chemorefractory mCRC. Based on our results, we suggested that combination therapy is much better than monotherapy. Furthermore, the efficacy of TAS-102 monotherapy and REG monotherapy were consistently similar. In our multivariate analysis, combination therapy were strong prognostic factors related with survival. These conclusions are clinical valuable and pave the way for the treatment of chemorefractory mCRC. Further prospective randomized controlled trials are warranted to validate our conclusions.

## Data Availability Statement

The raw data supporting the conclusions of this article will be made available by the authors, without undue reservation.

## Ethics Statement 

The studies involving human participants were reviewed and approved by E-Da Hospital Institutional Review Board (EMPR-109-012). Written informed consent for participation was not required for this study in accordance with the national legislation and the institutional requirements.

## Author Contributions

M-CH, K-MR, and H-PC wrote manuscript and performed clinical/genetic investigation. S-EL, K-WL, C-CC, C-IC, and L-CS performed clinical/genetic investigation. All authors contributed to the article and approved the submitted version.

## Conflict of Interest

The authors declare that the research was conducted in the absence of any commercial or financial relationships that could be construed as a potential conflict of interest.

## Publisher’s Note

All claims expressed in this article are solely those of the authors and do not necessarily represent those of their affiliated organizations, or those of the publisher, the editors and the reviewers. Any product that may be evaluated in this article, or claim that may be made by its manufacturer, is not guaranteed or endorsed by the publisher.
